# Mechanism of Paeoniflorin in the Treatment of Bile Duct Ligation-Induced Cholestatic Liver Injury Using Integrated Metabolomics and Network Pharmacology

**DOI:** 10.3389/fphar.2020.586806

**Published:** 2020-10-20

**Authors:** Shizhang Wei, Xiao Ma, Ming Niu, Ruilin Wang, Tao Yang, Dan Wang, Jianxia Wen, Haotian Li, Yanling Zhao

**Affiliations:** ^1^ College of Pharmacy, Chengdu University of Traditional Chinese Medicine, Chengdu, China; ^2^ Department of Pharmacy, PLA General Hospital, Beijing, China; ^3^ China Military Institute of Chinese Medicine, The Fifth Medical Center of PLA General Hospital, Beijing, China; ^4^ Department of Integrative Medical Center, The Fifth Medical Center of PLA General Hospital, Beijing, China

**Keywords:** paeoniflorin, cholestasis, bile duct ligation, metabolomics, network pharmacology

## Abstract

Paeoniflorin (PF) is the main active component of *Paeonia lactiflora* Pall., which is used in the treatment of severe cholestatic hepatitis. However, its biological mechanism in regulating bile acid metabolism and cholestatic liver injury has not been fully revealed. Our study aimed to reveal the mechanism of PF in the treatment of cholestatic liver injury in an *in vivo* metabolic environment using bioinformatics analysis. The serum of rats with bile duct ligation (BDL)-induced cholestatic liver injury treated with PF was analyzed by UHPLC-Q-TOF, and specific metabolites were screened using a metabolomics method. These specific metabolites were further analyzed by network pharmacology to identify the upstream signaling pathways and key protein targets. Finally, the key target proteins were verified by immunohistochemistry using cholestatic rat liver tissue. The serum ALT, AST, TBA, and TBIL levels, as well as the pathological state of the liver tissues, were significantly improved by PF. Twenty-five specific metabolites and 157 corresponding target proteins were screened for the treatment of cholestatic liver injury by PF. The “PF-target-metabolite” interaction network was constructed, and five protein targets (MAP2K1, MAPK1, ILBP, ABCB1, and LTA4H) that may regulate specific metabolites were obtained. The results of immunohistochemistry showed that PF improved the expression of these proteins. The integrated application of multiple bioinformatics methods revealed that PF plays a key role in the treatment of cholestatic liver injury by intervening in important targets related to bile acid metabolism and inflammation.

## Introduction

Cholestasis, which is mainly characterized by abnormal bile acid metabolism (generation, secretion, and excretion), is caused by various factors, such as alcohol (Roth and Qin, 2019), viruses (Hou et al., 2017), drugs (Chatterjee and Annaert, 2018), and autoimmunity (Trivedi and Hirschfield, 2016). Bile cannot flow into the duodenum and enter the blood circulation in the pathological state, and toxic bile acids accumulate in the liver. Chronic inflammatory responses, oxidative stress, mitochondrial abnormalities and other signals are activated, leading to damage to the liver and bile duct cells. Cholestasis is widespread, and patients show hyperbilirubinemia mainly because of the increase in direct bilirubin (DBIL), accompanied by jaundice, pruritus and fatigue. Continuous and severe cholestasis can progress to liver fibrosis, cirrhosis, hepatocellular carcinoma, and even liver failure (Pollock and Minuk, 2017). According to the pathological changes, cholestasis mainly manifests as hepatocellular and bile duct cholestasis. Clinically, primary biliary cholangitis (primary biliary cholangitis, PBC), drug-induced cholestasis, and primary sclerosing cholangitis (primary sclerosing cholangitis, PSC) are the most common presentations.

With the in-depth study of the pathogenesis of cholestasis, some key therapeutic targets have been successively identified: farnesoid X receptor (FXR); peroxisome proliferator-activated receptor (PPAR) (Beuers et al., 2015), which is involved in the regulation of bile acid metabolism; and the pregnane X receptor (PXR) (Teng and Piquette-Miller, 2007), which is involved in the regulation of liver detoxification. However, only a few drugs are available for treatment of cholestasis. The FXR agonist obacholic acid (OCA) is the first choice for the treatment of cholestasis (Kowdley et al., 2018) and can improve the condition of PBC patients who do not respond to ursodeoxycholic acid (UDCA). However, there are some clinical problems with OCA, such as dyslipidaemia, which increases the risk of cardiovascular disease (Nevens et al., 2016), and pruritus in 50% of patients with PBC cannot be significantly improved (Hirschfield et al., 2015). The screening and research of candidate drugs to treat cholestasis, such as the potential candidate drugs GW4064, GSK8062, and LY2562175 (Genin et al., 2015), which have been proven to be effective in the treatment of cholestasis in preclinical studies, are still being actively promoted. However, their clinical effects need to be investigated. Some compounds have certain toxic and side effects; for example, GW4064 has potential toxicity and slight instability. Therefore, it is important to identify safe and effective drugs for cholestasis based on effective methods.

Screening small-molecule natural compounds from traditional Chinese medicine (TCM) with strong clinical effects and good safety is an important method for research and development. PF is the main active component of *Paeonia lactiflora* Pall. and has been demonstrated to have a significant effect on the treatment of severe jaundice (He and Wang, 1997). Presently, the mechanism of PF in the treatment of cholestatic liver injury mainly involves antioxidative effects, antiapoptotic effects, and other mechanisms. However, no study has explored the molecular biological mechanism of PF in treating cholestatic liver injury using various bioinformatics methods.

**FIGURE 1:**
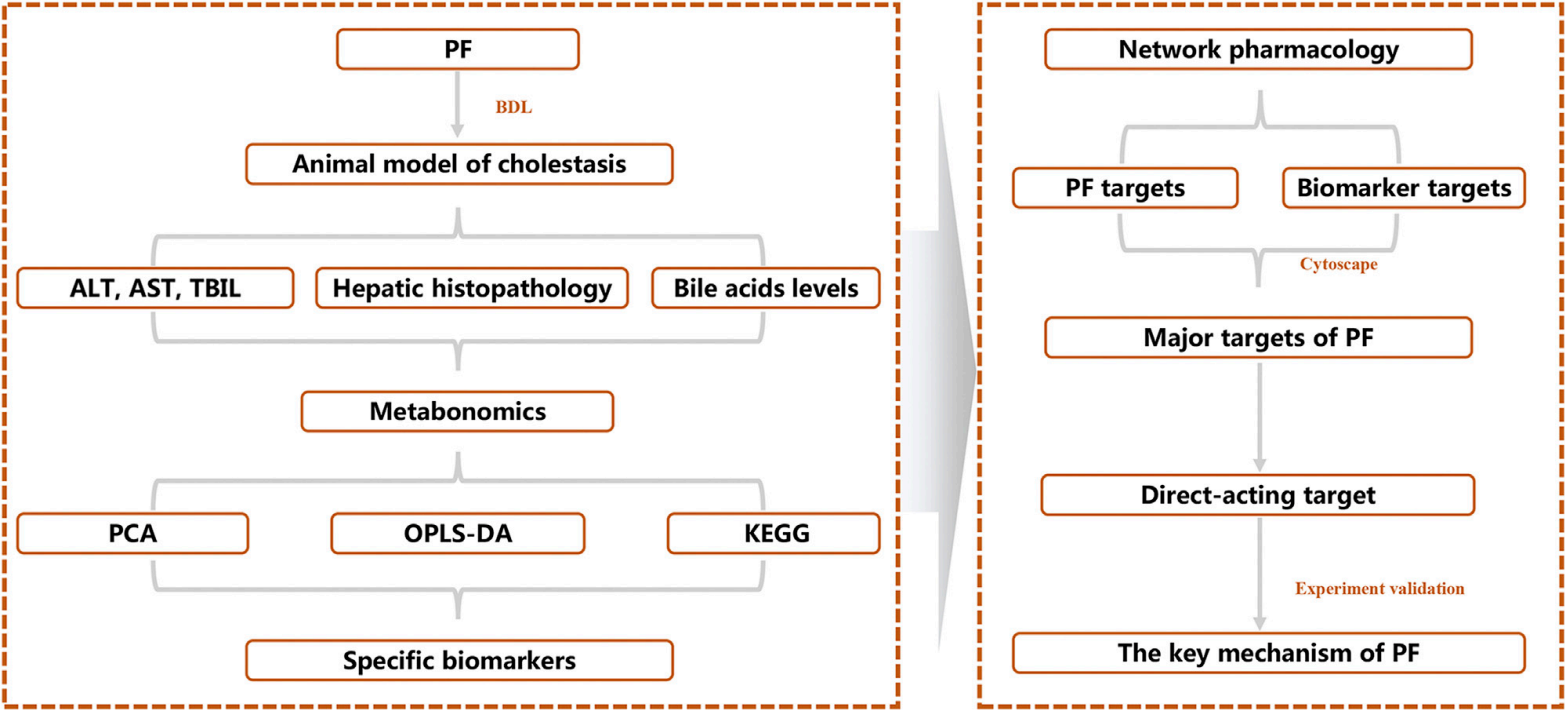
Schematic diagram of the molecular mechanisms of PF in treating cholestasis.

To reveal the therapeutic effect and molecular mechanism of PF on cholestasis, we integrated systematic pharmacological methods such as metabonomics, network pharmacology, and molecular biology to reveal the key targets of PF in cholestasis using the research mode of “PF-specific metabolite-target” from an *in vivo* metabolic environment of PF in the treatment of cholestasis (Figure 1).

## Materials and Methods

### Chemicals

PF was purchased from Chengdu Pufei De Biotech Co., Ltd. (Chengdu, China) and was shown to be more than 98% pure by ultraperformance liquid chromatography (UPLC). The alanine aminotransferase (ALT), aspartate transaminase (AST), total bilirubin (TBIL), and total bile acid (TBA) kits were purchased from Nanjing Jiancheng Bioengineering Institute (Nanjing, China). Dual-specificity mitogen-activated protein kinase kinase 1 (MAP2K1), mitogen-activated protein kinase 1 (MAPK1), ileal lipid-binding protein (ILBP), ATP-dependent translocase (ABCB1), and leukotriene A-4 hydrolase (LTA4H) antibodies were purchased from Beijing Yishan Biotechnology Co., Ltd. All biochemical indicator kits and other chemicals were commercially available.

### Animal Experimentation

Male Wistar rats weighing approximately 180 ± 20 g were obtained from the Laboratory Animal Center of the Military Medical Sciences Academy of the PLA (Permission No. SCXK-(jun) 2017-0010). All rats were maintained under a breeding environment (12 h:12 h light-dark cycle, 25 ± 0.5 °C, and 55 ± 5% relative humidity). The rats had free access to food and water. All animal experiments complied with the Ethics Committee of the Ethics of Animal Experiments of the Fifth Medical Center of PLA General Hospital (Approval ID: IACUC-2019-004).

After 1 week of acclimation, the animals were randomly divided into the normal, model, 200-mg/kg PF (PFH) and 50-mg/kg PF (PFL) groups, with six rats in each group. Bile duct ligation in the BDL, PFH, and PFL groups was performed according to standard methods (Pan et al., 2014). The rats in the normal group underwent bile duct mobilization but without bile duct ligation. On the second day after BDL operation, the rats in the normal and model groups were fed saline vehicle, and the rats in the PFH and PFL groups received a daily oral gavage of 200 and 50 mg/kg PF for 1 week, respectively. The rats were anesthetized with 20% ethyl carbamate solution, and blood samples were collected from the inferior vena cava to evaluate the biochemical indicators of liver function and metabolomics analysis. Next, the liver tissue was excised and fixed in 10% neutral-buffered formalin for HE staining.

### Analysis of the Biochemical Indicators of Liver Function

Rat serum centrifuged from the obtained blood was used to measure the levels of ALT, AST, TBIL, and TBA using commercial kits (Nanjing Jiancheng, Nanjing, China) according to the manufacturer’s protocol.

### Histological Examination

Histological examination was performed using 5-μm thick paraffin-embedded liver sections. Deparaffinized sections were stained with hematoxylin and eosin (H&E) as described previously (Wei et al., 2016).

### Immunohistochemistry

The 5-μm-thick paraffin-embedded liver sections were incubated with primary mouse monoclonal antibodies anti-MAP2K1, MAPK1, ILBP, ABCB1, and LTA4H for 90 min, followed by incubation with peroxidase-coupled secondary antibody for 30 min. Next, the sections were incubated with streptavidin-peroxidase-biotin complex for 20 min at room temperature. Color development was performed by incubation with 3,3′-diaminobenzidine tetrahydrochloride for 5 min, followed by visualization by light microscopy. The immunohistochemistry signals (positive areas) of MAP2K1, MAPK1, ILBP, ABCB1, and LTA4H were quantified using images captured with a digital camera system under ×200 magnification and analyzed by using Image-Pro Plus 6.0 (Media Cybernetics, MD, United States).

### Preparation of Samples for Injection

First, 200 μl of rat serum and 600 µl of methanol were transferred to a 1.5 ml centrifuge tube, mixed thoroughly with a vortexer, let stand for 20 min at 4 °C, and centrifuged at 12,000 rpm for 10 min at 4 °C. Then, the supernatant was transferred to a new 1.5 ml centrifuge tube and filtered through a syringe filter (0.22 μm) to obtain an injection sample.

### Preparation of Serum Samples for Metabolomics Analysis

The serum samples from the normal group, BDL group and PFH groups were subjected to LC/MS. Two hundred milliliters of serum samples from the three groups was treated as described previously (Ma et al., 2016) to obtain the samples for injection.

### Chromatography and Mass Spectrometry

The Agilent 1290 series UHPLC system was used for chromatographic analysis. All samples (4 μl) were detected using a Zorbax RRHD 300 SB-C18 column (2.1 mm × 100 mm, 1.8 mm; Agilent, United States) at 4 °C. The conditions of the mobile phase were consistent with our previous report (Wei et al., 2018). As a blank, the QC sample compounded with all samples was injected to guarantee the stability and repeatability of the UPLC-QTOF/MS systems after injection of the 10 samples. An Agilent 6550 Q-TOF/MS instrument (Agilent Technologies, Santa Clara, CA, United States) was used for mass spectrometry. The mass spectrometry analysis conditions were as follows: the electrospray capillary voltage was 3.0 kV in the negative ionization mode and 4.0 kV in the positive ionization mode; the gas temperature was 200 °C in the negative ionization mode and 225 °C in the positive ionization mode. The nozzle voltage was 500 V in both ionization modes; the gas flow was 11 L/min; and the mass range ranged from *m*/*z* 80 to 1,000.

### Data Extraction and Analysis

The sample data were extracted using MassHunter Profinder software. The MetaboAnalyst (last updated 2019-10-01) database was used to normalize the extracted data, and SIMCA-P 14.1 software (Umetrics, Umea, Sweden) was used for PCA (principal component analysis) and OPLS-DA (orthogonal partial least-squares-discriminant analysis) analyses. Significant variables (*p* < 0.05 in ANOVA, VIP > 1.5, and |p(corr)| ≥ 0.58) were selected as potential specific metabolites for further pathway enrichment analysis using the pathway analysis module in MetaboAnalyst.

### Identification of Paeoniflorin Targets and Potential Metabolites

PharmMapper Server (Version 2017) (Wang et al., 2017) was used to screen PF drug targets by importing the PF structure with the SDF format. The MBrole 2.0 (Version 2016) database was used to screen the corresponding targets of specific metabolites. The Database of Interacting Proteins (DIP) database was used to screen for interacting protein targets. All the ID types of the obtained targets were converted into UniProt IDs and were used to build the “PF-target-specific metabolite” interactive network using Cytoscape 3.7.2 (National Institute of General Medical Sciences, United States).

### Statistical Analysis

The data of the experiment were analyzed using the SPSS 13.0 software program. The differences among the experimental groups were calculated by ANOVA and are expressed as the mean ± SEM. *p* < 0.05 was considered statistically significant, and *p* < 0.01 was considered highly significant. SIMCA-P 14.1 software was used for pattern recognition analysis of PCA and OPLS-DA of serum metabolic compounds.

## Results

### Protective Effect of Paeoniflorin on Bile Duct Ligation Rats With Cholestatic Liver Injury

**FIGURE 2:**
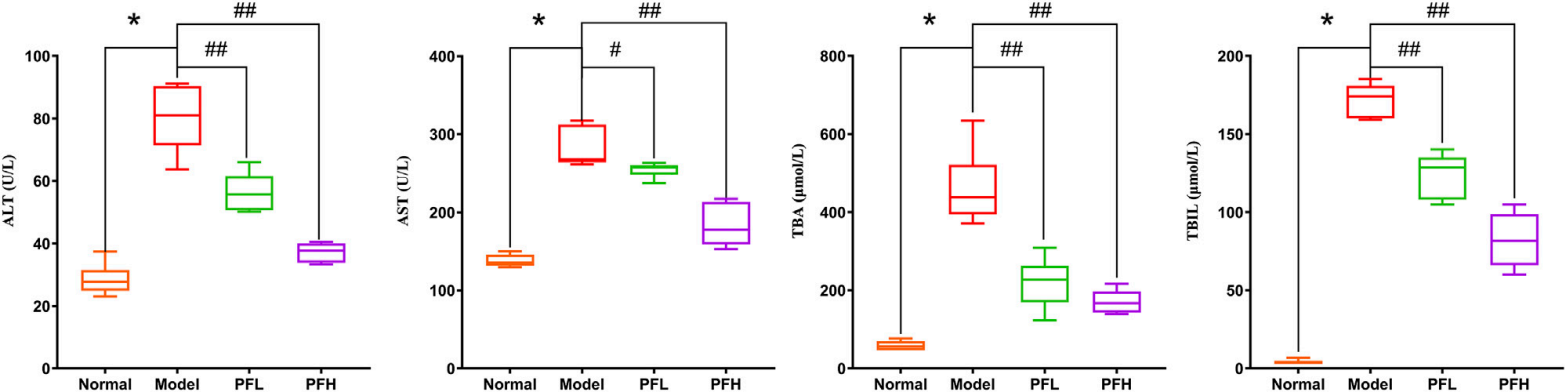
Effect of PF on the serum biochemical indicators (ALT, AST, ALP, TBIL, and TBA) of liver function. The data are expressed as the mean ± SEM. *p < 0.01 compared with the control group; ^#^ *p* < 0.05, ^##^ *p* < 0.01 compared with the model group.

**FIGURE 3:**
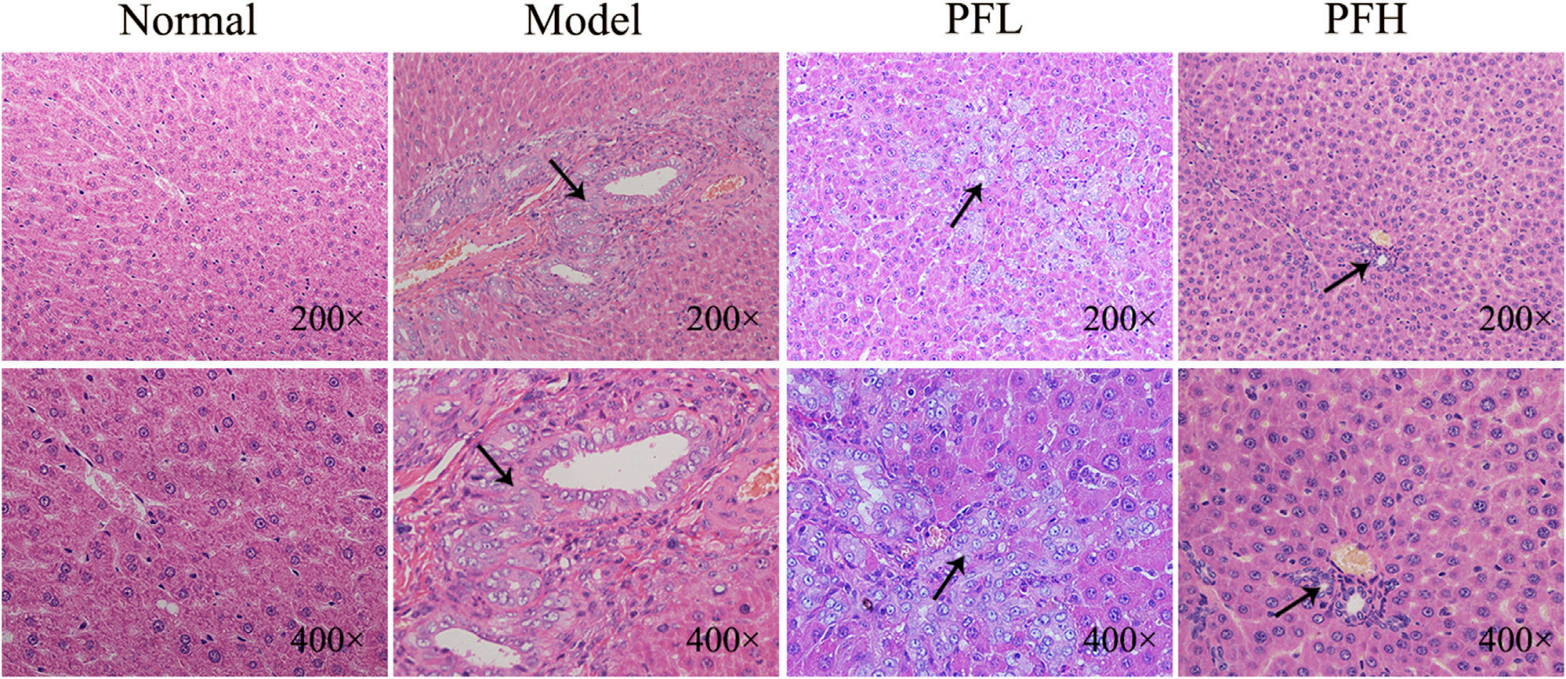
Effect of PF on the histopathology of liver tissues using HE staining (×200 and ×400).

To evaluate the effect of PF on cholestatic liver injury, we used BDL rats to induce the pathological state of cholestasis, and different doses of PF (200 and 50 mg/kg) were administered by gastric administration. The rat serum ALT, AST, TBIL, and TBA levels were evaluated. As shown in Figure 2, compared with those in the normal group, the serum levels of ALT, AST, TBIL, and TBA in the BDL model group were significantly increased. Compared with the model group treatment, PF (200 and 50 mg/kg) improved the elevation of these serum indicators. As shown in Figure 3, the results of HE staining indicated that the liver tissue of the rats in the BDL group showed spotty necrosis of hepatocytes, while PF (200 and 50 mg/kg) significantly improved the damaged liver tissue. These findings were similar to the results of the serum biochemical indexes.

### Multivariate Statistical Analysis

**FIGURE 4:**
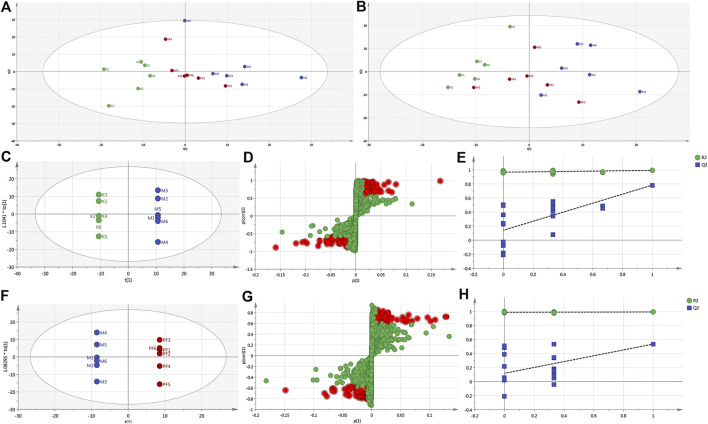
PCA score plot of the normal, BDL, and PF (200 mg/kg) groups in the ESI+ mode **(A)** and ESI− mode **(B)**. **(C)** Pairwise comparisons between the normal and BDL groups. **(D)** S-plots of the OPLS-DA model for the normal group and BDL group. **(E)** The 100-permutation test for the normal and BDL groups. **(F)** Pairwise comparisons between the BDL and PF groups. **(G)** S-plots of the OPLS-DA model for the BDL and PF groups. **(H)** The 100-permutation test for the BDL and PF groups. K, normal; M, model; PF, PFH.

To reveal the mechanism of PF in the treatment of cholestasis, we used metabolomics to detect the effect of PF on the metabolism of endogenous substances in the serum of the BDL rats. Principal component analysis (PCA) and orthogonal partial least squares discriminant analysis (OPLS-DA) in multivariate analysis were used to determine the endogenous substances that were significantly changed in the serum, as well as to screen out the key metabolic endogenous substances in the serum of cholestatic rats regulated by PF. PCA showed that under the anion and cation modes, the sample enrichment trends of the normal group, the BDL group, and the PF (200 mg/kg) dose group were distinguished (Figures 4A,B). The OPLS-DA results showed that the normal group, BDL group and PF (200 mg/kg) group were distinguished in both ion modes (Figures 4C,F; Supplementary Figure S1).

The R^2^X (cum), R^2^Y (cum), and Q^2^ (cum) of OPLS-DA in the positive model were 0.685, 1, and 0.832, respectively, using the data from the normal and BDL groups and 0.62, 1, and 0.571, respectively, using the data from the PF (200 mg/kg) and BDL groups. The R^2^X (cum), R^2^Y (cum), and Q^2^ (cum) of OPLS-DA in the negative model were 0.302, 0.979, and 0.678, respectively, using the data from the normal and BDL groups and 0.652, 1, and 0.376, respectively, using the data from the PF (200 mg/kg) and BDL groups. The analysis of the above parameters showed that the OPLS-DA model is of good quality and accurate.

In the positive (Figures 4D,G) and negative (Supplementary Figure S1) models, the variables with distant free dots were considered to contribute more to the separation between different experimental groups; thus, they were identified as specific metabolites. Permutation tests with 100 iterations were performed to verify the validity of the data multivariate analysis model. The results indicated that the original models are valid in the positive (Figures 4E,H) and negative modes (Supplementary Figure S1).

### Identification of Specific Metabolites of Paeoniflorin in the Treatment of Bile Duct Ligation-Induced Cholestatic Liver Injury

OPLS-DA analysis revealed endogenous metabolites with VIP ≥ 1.5 and |p(corr)| ≥ 0.58 from the normal, BDL, and PF groups (200 mg/kg) in the positive and negative modes that were screened as potential specific metabolites. Twenty-five specific metabolites were found to be related to treatment of cholestasis by PF and were mainly involved in bile acid metabolism (such as taurochenodeoxycholic acid, glycocholic acid, and urobinogen) and inflammation (such as N-acetyl-leukotriene E4 and leukotriene E4) (Table 1). The number of specific metabolites related to bile acid metabolism was greater, indicating that they were the main components in regulation of bile acid homeostasis by PF in the treatment of cholestasis, and they indirectly exerted anti-inflammatory effects during treatment for cholestatic liver injury.

**TABLE 1:** The specific metabolites of PF in the treatment of cholestasis.

No.	HMDB ID	Compound name	KEGG ID	Change trend	
				Model group^a^	PF group^b^
1	HMDB0000951	Taurochenodesoxycholic acid	C05465	↑	↓
2	HMDB0011662	3-Beta-hydroxy-4-beta-methyl-5-alpha-cholest-7-ene-4-alpha-carboxylate	C04840	↑	↓
3	HMDB0000138	Glycocholic acid	C01921	↑	↓
4	HMDB0001008	Biliverdin	C00500	↑	↓
5	HMDB0000698	Lithocholic acid glycine conjugate	C15557	↑	↓
6	HMDB0013626	Adrenoyl ethanolamide	C13829	↑	↓
7	HMDB0004158	Urobilinogen	C05791	↑	↓
8	HMDB0011637	Taurohyocholate	C15516	↑	↓
9	HMDB0001085	Leukotriene B4	C02165	↑	↓
10	HMDB0002178	3a,7a,12a-Trihydroxy-5b-cholestanoyl-CoA	C05448	↓	↑
11	HMDB0004158	Urobilinogen	C05790	↑	↓
12	HMDB0005084	N-Acetyl-leukotriene E4	C11361	↑	↓
13	HMDB0002012	Ubiquinone-1	C00399	↓	↑
14	HMDB0001023	4,4-Dimethylcholesta-8,14,24-trienol	C11455	↓	↑
15	HMDB0004085	Tuberculostearic acid	C16794	↑	↓
16	HMDB0005896	4-Hydroxyestradiol	C14209	↑	↓
17	HMDB0002200	Leukotriene E4	C05952	↑	↓
18	HMDB0000036	Taurocholic acid	C05122	↑	↓
19	HMDB0012639	20-Hydroxy-leukotriene E4	C03577	↑	↓
20	HMDB0002596	Deoxycholic acid 3-glucuronide	C03033	↑	↓
21	HMDB0000138	Glycocholic acid	C01921	↑	↓
22	HMDB0000036	Taurocholic acid	C05122	↑	↓
23	HMDB0011637	Taurohyocholate	C15516	↑	↓
24	HMDB0000086	Glycerophosphocholine	C00670	↑	↓
25	HMDB0002639	Sulfolithocholylglycine	C11301	↑	↓

a Change trends compared with the normal.

b Changes trend compared with the model group. The levels of differential metabolites were marked with downregulated (↓) and upregulated (↑).

**FIGURE 5:**
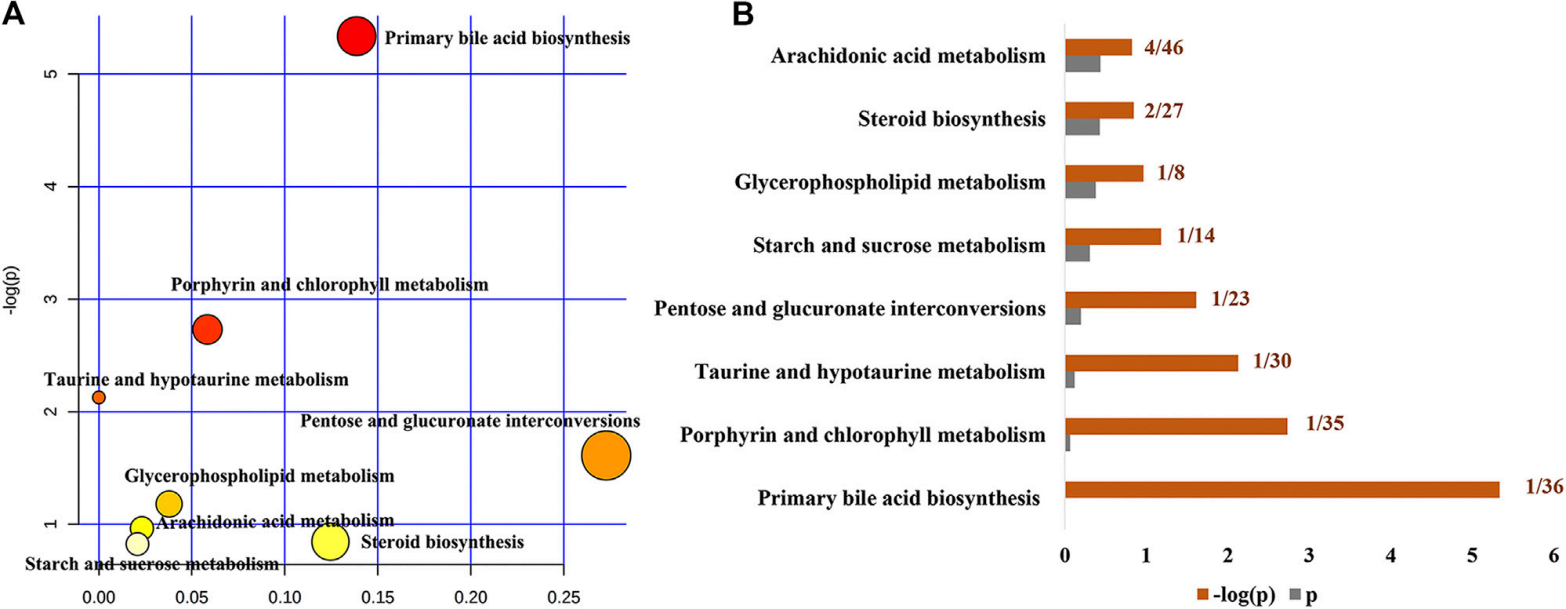
Pathway analysis of the specific metabolites of PF in the treatment of cholestatic liver injury. **(A)** Pathway impact by PF in the treatment of BDL-induced cholestasis. **(B)** Pathway details of PF in the treatment of BDL-induced cholestasis.

To further reveal the metabolic pathways of specific metabolites associated with the treatment of cholestasis by PF, we used the MetaboAnalyst database to determine enrichment by KEGG signaling pathways. Eight signaling pathways were enriched: primary bile acid biosynthesis, porphyrin and chlorophyll metabolism, taurine and hypotaurine metabolism, pentose and glucuronate interconversions, starch and sucrose metabolism, glycerophospholipid metabolism, steroid biosynthesis, and arachidonic acid metabolism (Figures 5A,B). Among them, primary bile acid biosynthesis is the most critical signaling pathway, suggesting that PF may play a therapeutic role in cholestasis by interfering with the primary bile acid metabolic pathway.

### “Potential Metabolite-Target-Component” Interactive Network and Analysis

**FIGURE 6:**
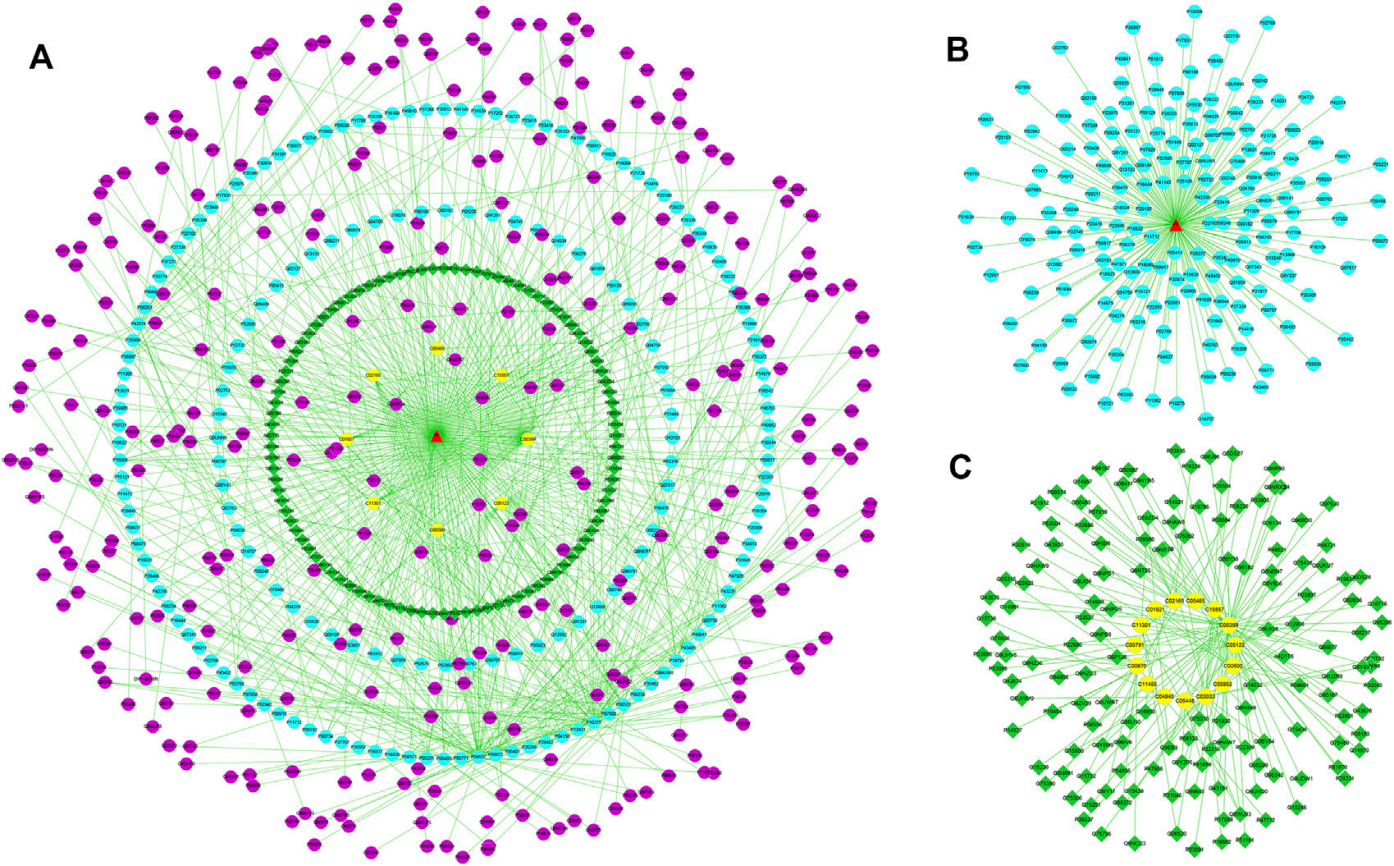
**(A)** The “specific metabolite-target-component” interactive network. **(B)** The “PF- target” interactive network. **(C)** The “specific metabolite-target” interactive network. The blue dots represent the protein targets of PF. The green squares represent the protein targets of specific metabolites. The purple dots represent interacting proteins. The yellow dots represent specific metabolites.

To reveal the probable upstream molecular biological mechanism by which PF regulates the above 25 specific metabolites, we used network pharmacology to analyze which targets PF interfered with to regulate the changes in specific metabolites. MBROLE 2.0 was used to collect specific metabolite-associated targets. One hundred eighty-two targets corresponding to 15 specific metabolites and 215 protein PF targets were identified. Cytoscape 3.7.0 software was used to visualize the network between PF and targets corresponding to specific metabolites. Nine target-specific metabolites, 182 PF targets, 108 specific metabolite targets, and 311 interacting proteins participated in the PF-target metabolite interaction network (Figures 6A–C).

**FIGURE 7:**
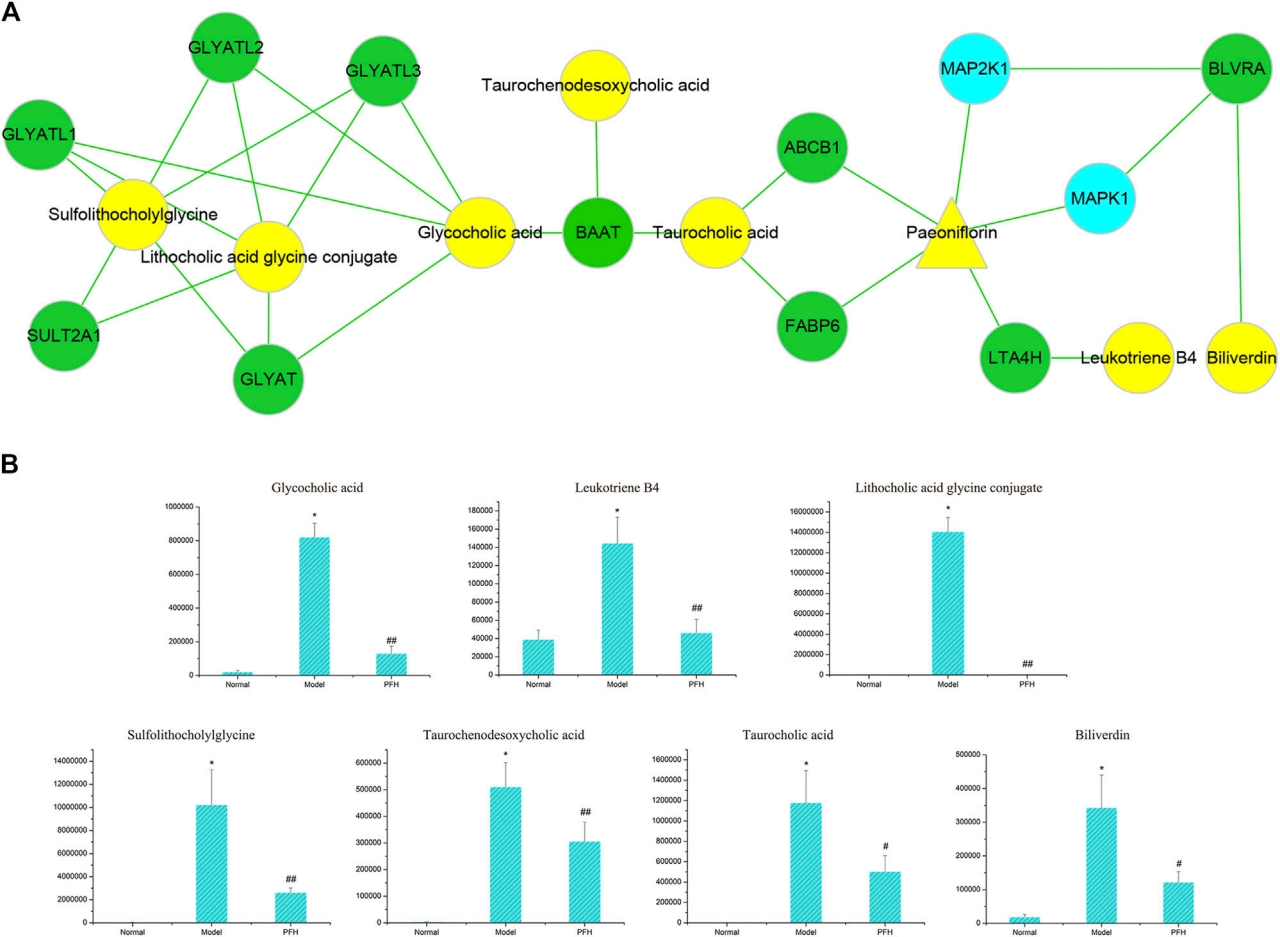
**(A)** The pivotal “specific metabolite-target-component” interactive network of PF in the treatment of cholestatic liver injury. The yellow triangles represent PF. The blue dots represent the protein targets of PF. The green squares represent the protein targets of specific metabolites. The yellow dots represent specific metabolites. **(B)** Changes in the metabolic level of the specific metabolites directly and indirectly regulated by PF. ^∗^ *p* < 0.01 compared with the normal group; ^##^ *p* < 0.01, ^#^ *p* < 0.05 compared with the BDL group.

PF directly regulated two target metabolites, leukotriene B4 (C02165) and taurocholic acid (C05122), by acting on fatty acid-binding protein 6 (P51161), ABCB1 (P08183), leukotriene a-4 hydrolase (P09960), and haem oxygenase 1 (P09601) (Figure 7A and Table 2). Four of the specific metabolites, taurochenodeoxycholic acid (C05465), glycocholic acid (C01921), lithocholic acid glycine conjugate (C15557), and sulfolithocholylglycine (C11301), were regulated indirectly. The changes in biliverdin (C00500) are indirectly regulated by Q02750 and P28482. Seven specific metabolites were significantly increased in the model group, but PF significantly decreased their expression (Figure 7B).

**TABLE 2:** The key direct targets of PF in the treatment of cholestatic liver injury.

No.	UniProt ID	Protein names	Gene names
1	Q02750	Dual specificity mitogen-activated protein kinase kinase 1	MAP2K1
2	P28482	Mitogen-activated protein kinase 1	MAPK1
3	P51161	Ileal lipid-binding protein	ILBP
4	P08183	ATP-dependent translocase	ABCB1
5	P09960	Leukotriene A-4 hydrolase	LTA4H

### Effect of Paeoniflorin on the Expression of Key Direct Targets Associated With the Treatment of Cholestatic Liver Injury

The key upstream molecular biological targets of PF in the treatment of cholestatic liver injury were investigated by combining metabolomics and network pharmacology analysis. The results showed that PF further affected endogenous metabolites (leukotriene B4, taurocholic acid, biliverdin, taurochenodeoxycholic acid, glycocholic acid, lithocholic acid glycine conjugate, and sulfolithocholylglycine), which were directly or indirectly related to MAP2K1, MAPK1, ILBP, ABCB1, and LTA4H.

**FIGURE 8:**
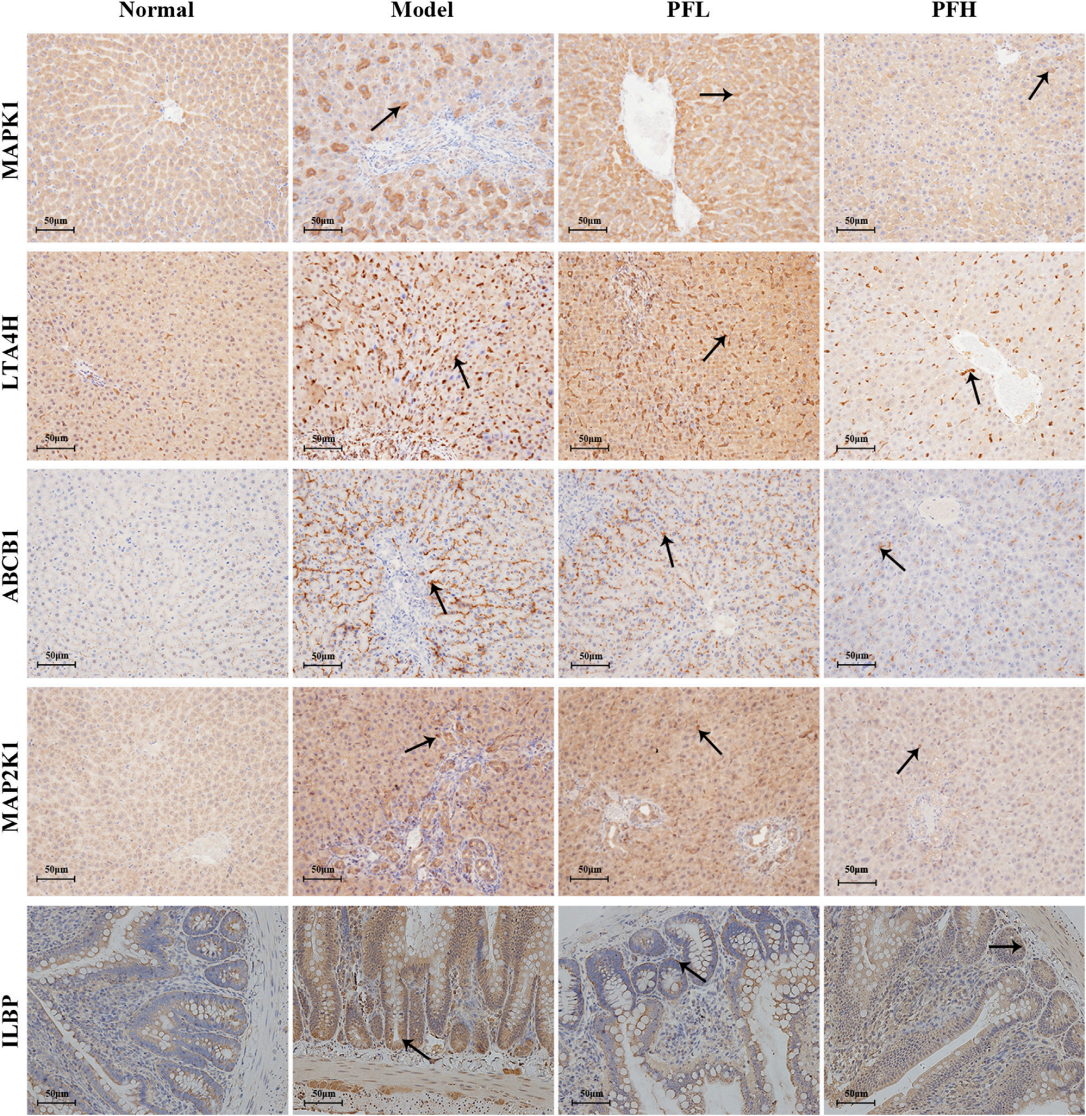
Effect of PF on the expression of MAPK1, LTA4H, ABCB1, MAPK2K1, and ILBP in the treatment of cholestasis detected by immunohistochemistry staining (×200). The key targets detected in liver tissue are MAPK1, LTA4H, ABCB1, and MAPK2K1, and the targets detected in the ileal tissue are ILBP.

**FIGURE 9:**
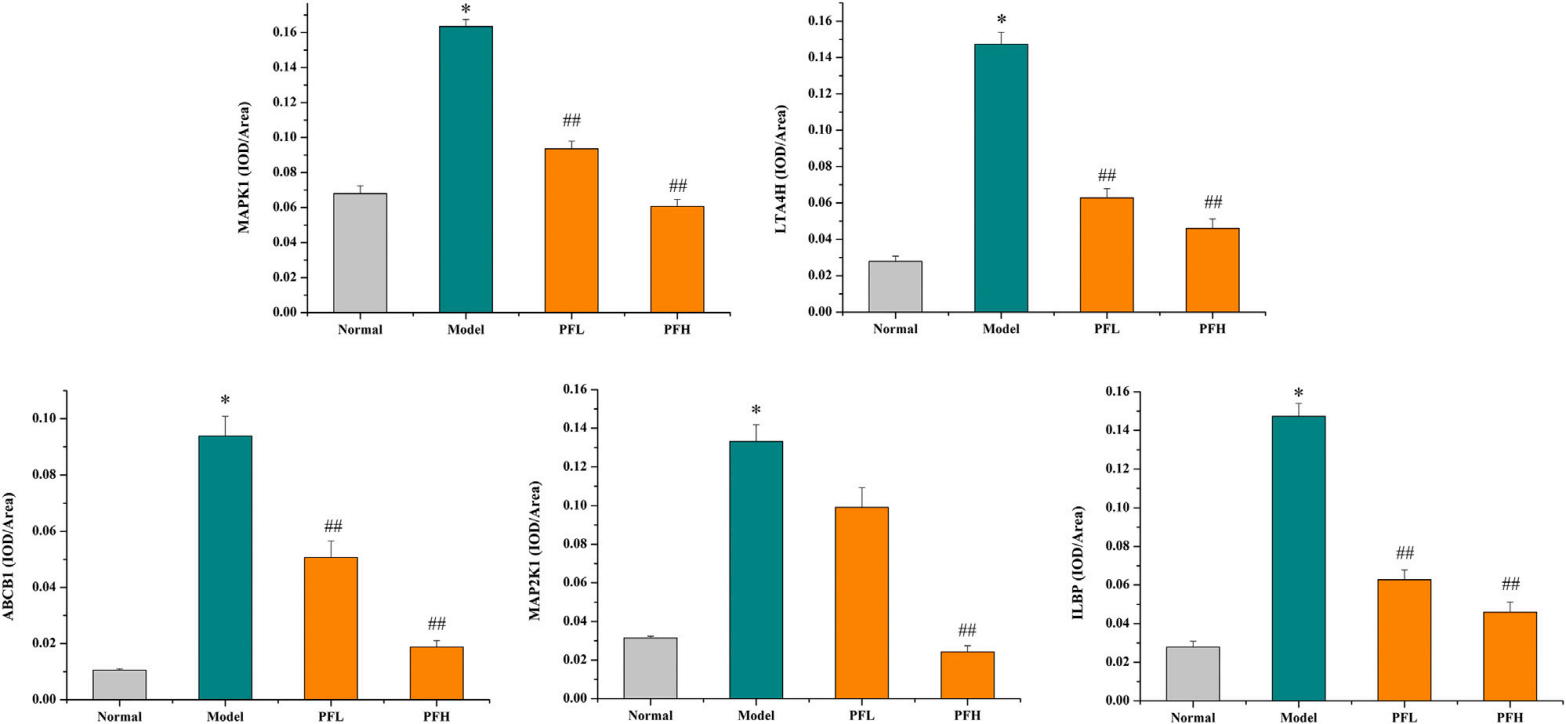
The effect of PF on the expression of MAPK1, LTA4H, ABCB1, MAPK2K1, and ILBP in the treatment of cholestasis detected by immunohistochemistry staining (×200). The data are expressed as the mean ± SEM, *n* = 3. ^∗^ *p* < 0.01 compared with the normal group; ^##^ *p* < 0.01, ^#^ *p* < 0.05 compared with the BDL group.

To verify the authenticity of the above results, we evaluated the effect of PF on the expression of the MAP2K1, MAPK1, ILBP, ABCB1, and LTA4H proteins by immunohistochemistry. As shown in Figures 8, **9**, compared with that in the normal group, the expression of MAP2K1, MAPK1, ABCB1, and LTA4H in the BDL group was significantly increased, and ILBP in the ileal tissue was significantly increased. However, the expression levels of the proteins in the PF groups (200 and 50 mg/kg) were significantly improved.

**FIGURE 10:**
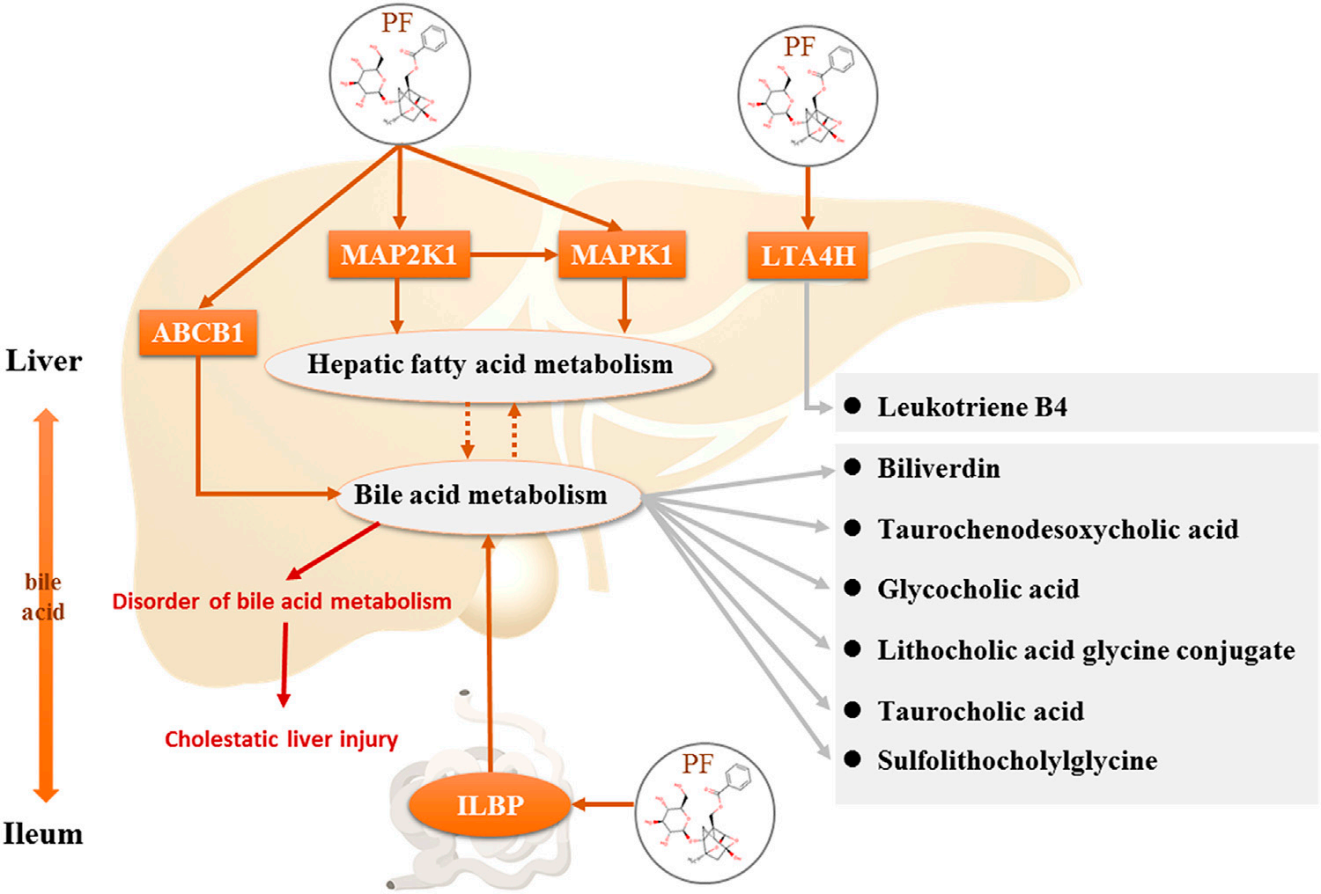
Direct targets of PF and the regulation of the corresponding metabolites in the treatment of cholestasis.

## Discussion

PF is the main active ingredient and the ingredient used for the quality control of *P. lactiflora* Pall. in Chinese pharmacopoeia. Previous studies have shown that PF can significantly improve cholestatic liver injury (Zhou et al., 2017), but the molecular mechanism of PF has not been revealed in an actual metabolic environment. This study first screened the specific metabolites of PF for the treatment of BDL-induced cholestatic liver injury using a metabolomics approach in an *in vivo* metabolite environment. Additionally, the regulation of these differentially abundant metabolites by PF was analyzed to clarify the therapeutic effect of PF on the pathological changes caused by cholestasis in the metabolic microenvironment. Among the specific metabolites screened out, the number of bile acid-related specific markers was the largest, followed by inflammation-related specific metabolites, suggesting that PF plays an important role in the treatment of cholestatic liver injury by improving bile acid metabolism and liver inflammation. However, the upstream signal targets that regulate the above specific metabolites are unclear. Therefore, to explore which key targets PF acts on and through which it regulates the above specific metabolites, other effective bioinformatics methods need to be applied.

Network pharmacological analysis methods have been increasingly used in drug development and molecular mechanism discovery. This method is also used to further assess the targets associated with specific metabolites regulated by PF. The key upstream targets regulating the above specific metabolites were analyzed and excavated using MBrole 2.0 (Version 2016). The results showed that PF regulated seven specific metabolites—leukotriene B4, taurocholic acid, biliverdin, taurochenodeoxycholic acid, glycocholic acid, lithocholic acid glycine conjugate, and sulfolithocholylglycine—through five targets (MAP2K1, MAPK1, ILBP, ABCB1, and LTA4H) in the interactive network. Studies have found that MAPK1 and MAP2K1 are two proteins involved in regulating fatty acid metabolism in the liver (Lawan and Bennett, 2017). ERK knockout mice are susceptible to dietary effects and exhibit liver steatosis (Khan et al., 2017), and the upregulation of the ERK signaling pathway may be related to liver function damage caused by steatosis (Jager et al., 2011). Cholesterol metabolism is closely related to bile acid metabolism, and the abnormal expression of the bile acid nuclear receptor farnesoid X receptor (FXR) is related to the homeostasis of bile acid metabolism and accumulation of triglycerides in the liver. In addition to the key targets of the classic signaling pathway of bile acid metabolism, FXR can also regulate cholesterol metabolism, fatty acid metabolism, and triglyceride metabolism through negative feedback (Van Rooyen et al., 2011; Bjursell et al., 2013). Moreover, the FXR-related factor apolipoprotein B also has a regulatory effect on cholesterol transport and metabolism (Li and Chiang, 2012). ILBP is a known cytoplasmic protein that binds and transports bile acids in the ileum and is probably the most important protein for bile acid absorption in intestinal epithelial cells (Kramer et al., 2001). Bile acids are synthesized in the liver and secreted by transporters such as BSEP into the small intestine (Gerloff et al., 1998). ILBP in the ileum reabsorbs and transports bile acids to the basement membrane, and these molecules are reabsorbed again by tASBT4 in the portal vein (Lee et al., 2006). The combined bile acid salts are taken up by NTCP in hepatocytes. The nonconjugated bile acid salts are taken up by hepatocyte OATPs and secreted into the bile ducts by the action of bile acid transporters such as MRP2 and BSEP, forming bile and further secretion. LTA4H is a zinc metalloenzyme associated with inflammatory diseases that can catalyze the biosynthesis of the proinflammatory mediator LTB4 to produce a proinflammatory response (Stsiapanava et al., 2014). The above findings revealed that PF regulates the enterohepatic circulation homeostasis of bile acids, thereby avoiding the accumulation of excess bile acids in the liver to cause toxicity to liver cells and liver damage. Additionally, by acting on LTA4H, a key target that triggers inflammatory responses, PF inhibits the inflammatory response caused by cholestasis and damages liver cells.

However, the discovery of the four upstream targets was only identified by computational analysis, and the results need to be further verified by animal experiments. Therefore, immunohistochemical staining was used to detect the expression of the five targets in the rat liver. The results showed that MAP2K1, MAPK1, ABCB1, and LTA4H were significantly increased in the BDL-induced cholestatic rats, ILBP was significantly increased in the ileal tissue, and PF significantly improved the expression of the above targets. The above findings revealed that PF can improve cholestatic liver injury via the following mechanisms: 1) regulating the bile acid transporter in the liver to dredge bile acid efflux and to avoid excessive accumulation of bile in the liver; 2) regulating biliary acid transport in the small intestine to avoid bile acid enterohepatic circulation disorders; and 3) improving the key proteins of the inflammatory response in the liver tissue to avoid an excessive inflammatory response (Figure 10). The above research results showed that PF can treat cholestasis through multiple pathways and multiple targets and can be used as a potential drug candidate for cholestatic liver disease.

## Conclusion

Our study demonstrated that PF is a promising candidate drug for the treatment of cholestatic liver injury by affecting key proteins in the bile acid transporter signaling pathway of the enterohepatic circulation of bile acids. These findings reveal that PF plays a multi-layered and multi-faceted role in the treatment of cholestatic liver injury from a mechanistic perspective.

## Data Availability Statement

The raw data supporting the conclusions of this manuscript will be made available by the authors, without undue reservation, to any qualified researcher.

## Ethics Statement

The animal study was reviewed and approved by Ethics Committee of the Ethics of Animal Experiments of the Fifth Medical Center of PLA General Hospital (Approval ID: IACUC-2019-004). Written informed consent was obtained from the owners for the participation of their animals in this study.

## Author Contributions

YZ participated in the design of the study. SW was responsible for primary data analysis and writing the manuscript. XM, MN, RW, TY, DW, JW, and HL were involved in the *in vivo* experimentation and technical work.

## Funding

This work is financially supported by grants from the National Natural Science Foundation of China (81874365) and Sichuan Science and Technology Program (2019YJ0492).

## Conflict of Interest

The authors declare that the research was conducted in the absence of any commercial or financial relationships that could be construed as a potential conflict of interest.
