# Supplementary material for: Mechanism of Paeoniflorin in the Treatment of Bile Duct Ligation-Induced Cholestatic Liver Injury Using Integrated Metabolomics and Network Pharmacology
Source: Front Pharmacol. 2020 Oct 20;11:586806. doi: 10.3389/fphar.2020.586806 (PMC7641625; doi:10.3389/fphar.2020.586806)
Supplement: Supplementary file 1 [file Image1_v1.pdf]

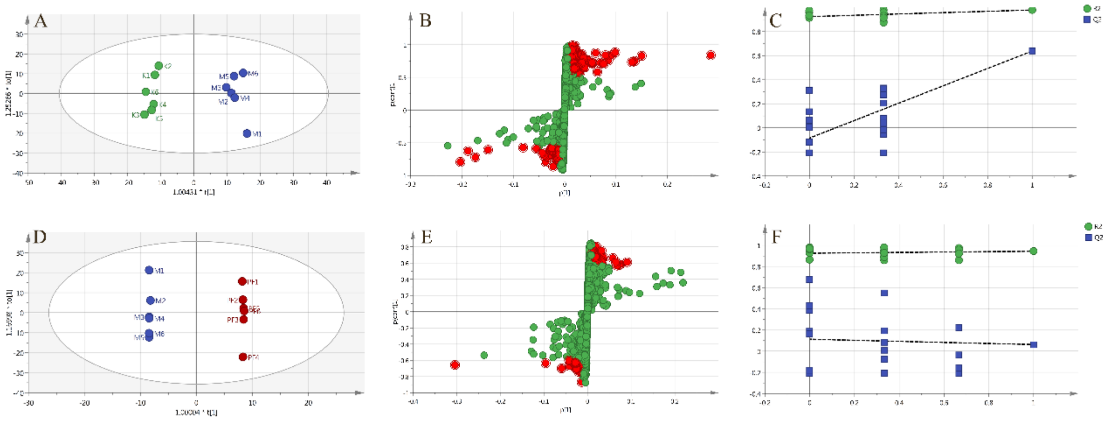

FIGURE S1 The OPLS-DA score plots, S-plots and 100-permutation test generated from the OPLS-DA data of the normal, model and PF groups in the ESI- mode. A: The pair-wise comparisons between the normal and model groups; B: The S-plots of the OPLS-DA model for the normal and model groups; C: The 100-permutation test for the normal and model groups; D: The pair-wise comparisons between model and PF groups. E: The S-plots of the OPLS-DA model for the model and PF groups. F: The 100-permutation test for the model and PF groups.
